# Path following Control of an Underactuated Catamaran for Recovery Maneuvers

**DOI:** 10.3390/s22062233

**Published:** 2022-03-14

**Authors:** Sang-Do Lee, Yong-Seung Song, Dae-Hae Kim, Ma-Ru Kang

**Affiliations:** 1 Division of Navigation & Information System, Mokpo National Maritime University, Mokpo 58628, Korea; oksangdo@mmu.ac.kr; 2 Korea e-Navi Information Technology Co., Ltd., Busan 49111, Korea; vmsongys@naver.com (Y.-S.S.); mail2hae@naver.com (D.-H.K.); 3 Department of Defense Science & Technology, Gwangju University, Gwangju 61743, Korea

**Keywords:** recovery maneuver, underactuated, error dynamics, path following control, catamaran, rate of turn, adaptive backstepping

## Abstract

This paper focuses on the autonomous recovery maneuvers of an unknown underactuated practical catamaran, which returns to its initial position corresponding to the man overboard (MOB) by simply adjusting the rate of turn. This paper investigates the completion of model-based path following control for not only the traditional Williamson turn, but also complex recovery routes under time-varying disturbances. The main difficulty of model-based path following control for predicting the hydrodynamic derivatives of a practical catamaran was solved by the approximated calculation of a diagonal matrix. The second key problem of differential calculation for an underactuated model in the case of complex reference trajectories under severe disturbances was investigated. Even though this paper employs a diagonal matrix with unknown nonlinear terms, the experimental test using a small craft with payloads by remote control demonstrated the sway force per yaw moment in turning cases. Adaptive backstepping mechanisms with unknown parameters were proven by the Lyapunov theory as well as the passive-boundedness of the sway dynamics, guaranteeing the stability of sway motion in the case of unavailable sway control. The effectiveness of the algorithms of the guiding concept and error dynamics is demonstrated by the numerical simulations.

## 1. Introduction

Path following control has been broadly addressed in the motion control of autonomous vehicles. Motion control scenarios of autonomous vehicles are usually divided into three or four categories, such as setpoint stabilization, trajectory tracking, path following, or target following [1,2,3]. Since there are countless publications related to motion control scenarios for the past decades, it is not easy to understand the key technologies. Thus, there is a need to pinpoint essential skills such as underactuated and error dynamics.

Most of all, the concept of “underactuated” plays a very important role in path following control, especially in marine vehicles. Motion control systems are implemented to control the motion of unmanned aerial/underwater vehicles and unmanned surface crafts via actuators [4]. In this paper, underactuated systems having fewer actuators than the actual number of degrees-of-freedom (DOF) of the system will be considered to solve the path following problem [5]. For a marine surface vehicle, DOF equals the set of independent displacements and rotations that completely specify the displaced position and orientation of the vehicles [6]. Therefore, an underactuated system means that a marine vehicle has fewer control inputs than the number of generalized coordinates [6].

Most marine surface vehicles are underactuated, since they cannot produce control forces and moments in all DOFs [6]. Thus, they are equipped with screw propellers at a distance from the centerline [7] for surge force control and various kinds of rudders for yaw moment control. Therefore, there is no direct control actuator for sway motion in the underactuated marine vehicles. State-of-the-art actuation systems such as tunnel thrusters, podded drive system, or cycloidal (Voith-Schneider or vertical) propellers are not effective with respect to sway motion at high speeds [8].

As mentioned earlier, underactuated systems have fewer numbers of actuators than the actual number of DOFs to be controlled [9], and include nonintegrable constraints on acceleration in nonholonomic systems [10], which is not transformable into a driftless chained form [11]. Since underactuated systems cannot be asymptotically stabilized by a feedback control law, this problem is not solvable using feedback linearization [7]. To tackle the inherent nonlinearity in underactuated ship dynamics or path following kinematics [11], robust nonlinear control methods have been investigated over the last few years. In this paper, an adaptive backstepping method [12] will be employed to solve the path following problem.

In integrator backstepping, one can see the complexity of the explosion term in calculating the reference state [13]. This well-known “explosion of complexity”, which is caused by repeated differentiation of nonlinear functions in a virtual control [14,15], can be solved by dynamical surface control (DSC), which uses an auxiliary first-order low-pass filter at each backstepping step [8,13].

In addition, error dynamics is the most fundamental skill for solving any problem of autonomous operation such as path following, automatic berthing, and collision avoidance. The theory of error dynamics has been broadly employed in controls. This skill is frequently used in backstepping controllers [11], trajectory tracking, and dynamics positioning [16]. During the whole path following process, motion errors exist between the virtual ship and the own (or actual) ship. The guiding principles handle the error variables in a closed-loop system. To converge the variables (trajectories) to an invariant set [11] or equilibrium, the control system should be defined based on the error dynamics.

Meanwhile, recovery maneuvers [17] are an essential skill for saving human lives in emergencies. Even though seafarers regularly perform the legal rescue training for retrieving a man overboard (MOB), it is difficult to return to the original track lines using conventional recovery maneuvers such as the Scharnov turn [18], the Williamson turn [19], and the Anderson turn [20]. Practically, regardless of sea state, it is very difficult for a duty officer to find a survivor on the sea even if the ship returns to the exact point of casualty. Nevertheless, to save human lives in distress, marine vessels should return to the initial position as much as is possible under the weather conditions.

For a traditional ship, the officers give sequential orders of rudders corresponding to the standard recovery maneuvers [17,20]. However, this requires skillful rudder action in order to eventually succeed in the mission. Moreover, this approach seems to be ineffective for unmanned surface vehicles (USV), since they may have various propulsion systems equipped not limited to the single rudder. Therefore, this paper focuses on the recovery maneuvers performed by a USV to return to the initial point by simply adjusting the rate of turn as well as the completion of the model-based path following control.

However, to the best of our knowledge, many reports of model-based path following control are limited to the simple routes (straight line [3], curved line [5,21], port [8,22,23] and starboard turning [9,11]) with an existing model presented in [2,3,5,8,9,11,22,23,24] that includes the unknown restoring forces in the dynamics. Conversely, some numerical publications [25,26,27,28] without experiments handle the non-diagonal matrices for sway/yaw added mass ($m_{23}$ of 3DOF system, A26 of 6 DOF system) for practical situations based on port/starboard symmetry hull forms; however, this overlooks the nonlinear restoring terms in the dynamics, because the fast convergence and robust stability of the underactuated model considering both unknown nonlinear restoring terms and $m_{23}$ are more challenging problems. Otherwise, no specific dynamics or hydrodynamic coefficients can be seen in experiments using USV [29].

In any case, the main difficulty of model-based path following control is to predict the hydrodynamic derivatives due to the acceleration in the directions of the surge, sway, and yaw motions, as well as to solve the differential problems calculating an unknown underactuated practical model under external disturbances. Motivated by these problems, this paper investigates the following key contributions and draws comparisons with the existing works in the literature.

(1) A model-based approach of path following control with unknown dynamics was built based on the previous numerical scheme by Lee [24] using a practical underactuated catamaran model. Adaptive backstepping [8,11,12], guiding principles for reference trajectories [8,11,22,23], and DSC skill [8,14] were applied to the practical model in this paper.
(2) A real catamaran was prepared to check the maneuvering conditions in both straight and turning situations by remote control inshore. Marine GPS sensors continuously received information of the latitude/longitude position, speed over ground, ground heading, etc. Unfortunately, in this paper, both straight and turning line tracking algorithms of the LOS type [3,21], including side slip angle, were not adopted in the experimental test because non-diagonal matrices for $m_{23}$ should be considered in practice. However, we favor the diagonal system, which can represent the hydrostatic forces and restoring force of a catamaran. Additionally, this element of a positive inertia matrix is much smaller than the diagonal counterparts [6,16].
(3) The main purpose is to solve path following control in case of recovery maneuvers for an unknown underactuated practical catamaran. We address the possible drawbacks of the traditional Williamson method via the turning rate of the USV, rather than rudder usages. This facilitates returning to the initial spot of the MOB more accurately. Additionally, a complex route including successive opposite turning under disturbance is defined. This new result overcomes the differential problems of an underactuated model in the case of complicated reference trajectories. This demonstrates incremental progress compared to the restricted trajectories in many previous papers, which attempt to achieve fast convergence and robust stability [30] for their control development.

This study focuses on returning to the initial point of casualties by means of the ship’s turning rate. This paper highlights that the practical catamaran returns to the departure point (MOB) as much as possible in the case of complicated routes and disturbances. Additionally, DSC skills will be implemented to overcome the “explosion of complexity” problem. The guiding principles and adaptive back-stepping methods of the underactuated catamaran have been verified by some numerical simulations under disturbances.

The rest of the paper is structured as follows. Section 2 briefly introduces the underactuated catamaran with a 3DOF system and describes the path planning and adaptive backstepping controller design. In Section 3, the system stability analysis using Lyapunov theory and the passive-boundedness of sway motion will be introduced and proven mathematically. In the discussion, the numerical simulations will be shown to demonstrate the effectiveness of the proposed control scheme. Finally, conclusions based on this research will be briefly addressed.

## 2. Materials and Methods

### 2.1. Problem Formulation

#### 2.1.1. Notations

In this paper, ${ℜ}^{n}$ is n-dimensional Euclidean space. | ⋅ | represents the absolute value of a scalar ( ⋅ ), while ‖ ⋅ ‖ means the Euclidean norm of a vector or the Frobenius norm of a matrix [3]. $\overset{\wedge }{\left(\;\cdot \;\right)}$ is the estimation of ( ⋅ ) and $\tilde{\left(\;\cdot \;\right)}$ = $\overset{\wedge }{\left(\;\cdot \;\right)}\;-\left(\;\cdot \;\right)$ denotes its estimated errors.

#### 2.1.2. Underactuated Catamaran Model

**Figure 1:**
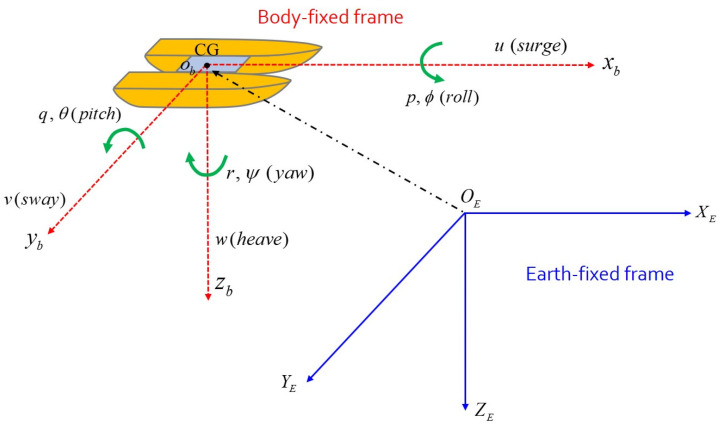
Coordinate systems (body-fixed frame and earth-fixed frame).

The motions of the catamaran are considered on a horizontal plane. The horizontal motion is defined in the surge, sway, and yaw directions as shown in Figure 1 [21]. In this paper, the body-fixed frame and the earth-fixed frame $O_{E}X_{E}Y_{E}Z_{E}$ are considered. The origin $o_{b}$ of the body-fixed frame is a moving coordinate frame [10] and is located at the center of gravity (CG). The body axes $x_{b},\;y_{b}$, and $z_{b}$ are selected to coincide with the principal axes of inertia for the catamaran. The $x_{b}$ is a longitudinal axis (directed from aft to fore) and $y_{b}$ represents a transverse axis (directed to starboard). Lastly, $z_{b}$ means the normal axis (directed from top to bottom) [1].

The coordinates (x, y, z) are equivalent to the position and translational motion of the catamaran, while the coordinates (ϕ, θ, ψ) mean the rotational motion about the x, y, and z axes, respectively [6]. The state vectors are given as $\eta ={\left[x,\;y,\;\psi \right]}^{T}$ for the position and orientation vector, and $\upsilon ={\left[u,\;v,\;r\right]}^{T}$ for the linear and angular velocity vector, where (x, y, ψ) denotes the coordinate of catamaran’s position and yaw angle about the earth-fixed frame. Consequently, heave (w), roll (p), and pitch (q) motions are neglected. This means that the dynamical motions of the catamaran will be limited to a horizontal plane [24].

The kinematics can be reduced from the general 6DOF to 3DOF expression. In addition, it is assumed that the catamaran can be considered a homogeneous mass distribution with an xz plane of symmetry such that $I_{xy}$ = $I_{yz}$ = 0 [6]. With these assumptions, the dynamical motions of the catamaran moving in a horizontal plane can be simply expressed as

(1a) $$\left[\begin{array}{c} \dot{x}\\ \dot{y}\\ \dot{\psi } \end{array}\right]=\left[\begin{array}{ccc} \cos\psi & -\sin\psi & 0\\ \sin\psi & \cos\psi & 0\\ 0 & 0 & 1 \end{array}\right]\left[\begin{array}{c} u\\ v\\ r \end{array}\right]$$

(1b) $$\left[\begin{array}{c} \dot{u}\\ \dot{v}\\ \dot{r} \end{array}\right]=\left[\begin{array}{c} W_{u}^{T}\cdot f_{u}(\dot{\eta },\;\eta )\\ W_{v}^{T}\cdot f_{v}(\dot{\eta },\;\eta )\\ W_{r}^{T}\cdot f_{r}(\dot{\eta },\;\eta ) \end{array}\right]+\left[\begin{array}{cc} \frac{1}{m_{11}} & 0\\ 0 & 0\\ 0 & \frac{1}{m_{33}} \end{array}\right]\left[\begin{array}{c} \tau_{u}\\ \tau_{r} \end{array}\right]+\left[\begin{array}{ccc} \frac{1}{m_{11}} & 0 & 0\\ 0 & \frac{1}{m_{22}} & 0\\ 0 & 0 & \frac{1}{m_{33}} \end{array}\right]\left[\begin{array}{c} d_{wu}(t)\\ d_{wv}(t)\\ d_{wr}(t) \end{array}\right]$$

where $W_{u}\in \;{ℜ}^{n_{u}}$, $W_{v}\in \;{ℜ}^{n_{v}}$, $W_{r}\in \;{ℜ}^{n_{r}}$ represent the unknown constant vectors having the known dimensions $n_{u},\;n_{v}$ and $n_{r}$; $f_{u}(\dot{\eta },\;\eta )\in \;{ℜ}^{n_{u}}$, $f_{v}(\dot{\eta },\;\eta )\in \;{ℜ}^{n_{v}}$ and $f_{r}(\dot{\eta },\;\eta )\in \;{ℜ}^{n_{r}}$ are the known smooth vector fields; $\tau_{u}$ and $\tau_{r}$ are the actual inputs such as propulsion force and yaw moment with the known nonzero constant control coefficients $1/m_{11}$ and $1/m_{33}$; $d_{wu}$, $d_{wv}$, and $d_{wr}$ denote the unknown time-varying disturbances of forces and moment [11,22,24].

**Remark** **1.** *During the recovery maneuvers, the environmental disturbances play an important role. However, it is not easy to define the exact amounts of time-varying environmental disturbances on the practical spot in distress. In general, ocean environmental disturbances can be considered as all possible aspects of sailing conditions, such as waves, winds, currents, ice-covered waters, water on deck by slamming, ship-to-ship interaction forces in close proximity [31,32,33], or transversal exciting forces [34,35,36]. The USV should return to the initial point under any sea circumstances*.

**Assumption** **1.** *Time-varying environmental disturbance $d_{wi}$ is assumed to be bounded by unknown constants $d_{wu\max}$, $d_{wv\max}$, and $d_{wr\max}$, that is, $\left|d_{wu}\right|\leq d_{wu\max}$, $\left|d_{wv}\right|\leq d_{wv\max}$, $\left|d_{wr}\right|\leq d_{wr\max}$ [8,11,21,22,23,24]*.

**Remark** **2.** *As for the conventional ship system, a routine order will be given to the rudders for the standard recovery maneuvers. In general, the turning rate of the catamaran reaches a turning circle of 540 degree with a constant rudder angle. Thus, there is a rudder execute time in the turning circle information. However, recent USVs are not limited to a single rudder system only, but rather are equipped with unnecessary rudder systems such as podded propeller types (Azipod, Mermaid, etc.), unfixed twin propellers, and cycloidal (Voith-Schneider or vertical) propeller [37]. Thus, the control input $\tau_{r}$ is considered to produce the total amount of yaw moment by means of any type of rudder or actuation system. Thus, a specific rudder machinery model is not presented in the control scheme [24]*.

**Remark** **3.** *When the duty officers become aware of the side of MOB, they perform a kick action with hard-over at first. However, this concept is based on the crew on the ship. If there are no watchmen on the deck or observers inshore, it is difficult at first to recognize the side of the men in distress. Therefore, we consider that the initial point of the USV is the position of the MOB. When the USV returns to the initial point as nearly as possible despite sea conditions, the recovery maneuvers are successful*.

**Assumption** **2.** *The recovery routes of own catamaran are prescribed by the virtual catamaran*

(2a) $${\dot{x}}_{d}=u_{d}\cos(\psi_{d})\;\;$$

(2b) $${\dot{y}}_{d}=u_{d}\sin(\psi_{d})\;$$

(2c) $${\dot{\psi }}_{d}=r_{d}\;$$

*where the reference $\eta_{d}={\left[x_{d},y_{d},\psi_{d}\right]}^{T}$ and all of its first- and second-order derivatives ${\dot{\eta }}_{d},\;{\ddot{\eta }}_{d}$ are all bounded [8,11,22,23,24]. The recovery path is mainly affected by the turning rate of virtual catamaran ($r_{d}$) and constant speed ($u_{d}$)*.

**Assumption** **3.** *The sway velocity (v) in Equation (1b) is stable under passive-boundedness*.

**Definition** **1.** *Consider a system* $x_{i}=f(\vec{x})+d$ *, where* $\vec{x}={\left[x_{1},\;\ldots \;,x_{i}\right]}^{T}$ *,* i=1, …, n *denotes input vectors.* $f(\vec{x})={ℜ}^{n}\to ℜ$ *is an unknown function and* d *is a disturbance term. For all bounded* $x_{j},\;j\neq i$ *and* d *, if there exists a Lyapunov function* $V(x_{j}),\;\in \;C^{i}$ *such that*

*(1)* 
$V(x_{i})$ *is globally positive definite and radially unbounded,*
*(2)*  $\dot{V}(x_{i})<0$*if*$\left|x_{i}\right|>x_{i}^{*}$, where $x_{i}^{*}$is a positive constant and related to the bounds of $x_{j},\;j\neq i$*and*d.
  *We define the state variable*$x_{i}$*is stable under passive-boundedness [8,11]*.

### 2.2. Path Planning and Controller Design

#### 2.2.1. Recovery Path Planning

Guidance systems in marine vessels are used to generate a predefined path for time-invariant path following. The guidance represents the basic methodology concerned with the transient behavior associated with the achievement of motion control objectives.

**Figure 2:**
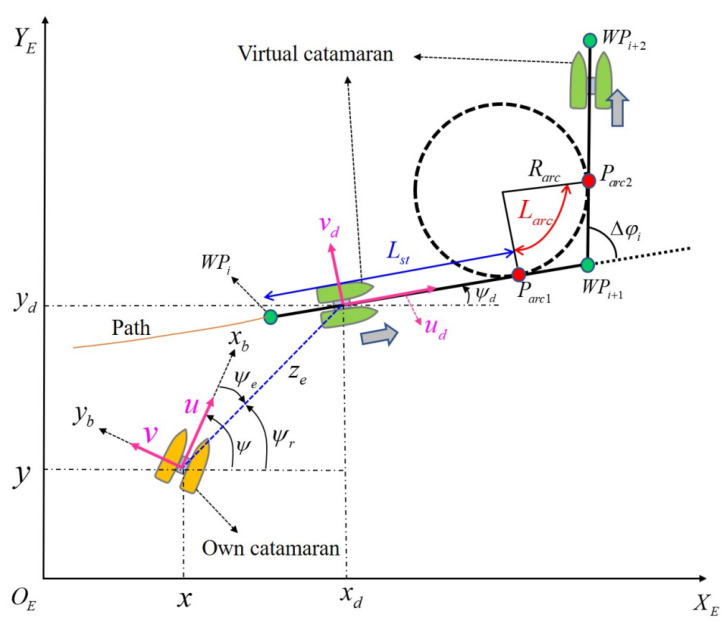
Guiding principles and a framework of path following control between the own catamaran and the virtual catamaran.

The differences between a conventional ship and a USV with respect to recovery maneuvers are in path planning. As stated in Assumption 2, the recovery path is highly dependent on the turning rate of the virtual catamaran ($r_{d}$) and the constant speed ($u_{d}$). Therefore, the recovery path should be defined using both $r_{d}$ and $u_{d}$. Figure 2 shows recovery path planning with two catamarans. If a virtual (ideal) catamaran makes an original path, then the USV follows the virtual catamaran. The USV moves along the predefined path regardless of the position of MOB. In Figure 2, a virtual catamaran on the straight ($L_{st}$) goes forward with constant speed ($u_{d}$), and its corresponding time $t_{s}=L_{st}/u_{d}$. The recovery path is generated by the virtual catamaran starting at a certain point ($WP_{i}$). After the catamaran goes toward the next point ($WP_{i+1}$), it will lie on the arc line from a first curved point ($P_{arc1}$) and will arrive at the final curved point ($WP_{i+2}$). Then, the angle of $WP_{i}\;WP_{i+1}$ can be defined as

(3) $$\phi_{i}=\arctan\frac{y_{i}-y_{i-1}}{x_{i}-x_{i-1}}\;$$

where $\phi_{i}$ is the angle of the recovery path, that is, the heading angle of the virtual catamaran. When the virtual catamaran changes its course to port and starboard with turning rate ($r_{d}$), the recovery path shifts from a straight line to a curved line ($L_{arc}$). Therefore, the virtual catamaran passes through the curved points ($P_{arc1}$, $P_{arc2}$). In addition, the real-time turning radius ($R_{arc}$) can be determined by the turning rate ($r_{d}$) with interpolation in ($R_{\min}$, $R_{\max}$) as follows [23,24].

(4) $$R_{arc}=\{\begin{array}{cc} R_{\max} & if\;\left|\Delta \phi_{p}\right|>\pi /2\\ \frac{(R_{\max}-R_{\min})\Delta \phi_{p}}{sign(\Delta \phi_{p})\frac{\pi }{2}} & if\;\left|\Delta \phi_{p}\right|<\pi /2 \end{array}\;$$

where $\Delta \phi_{p}$ denotes the practical changes of heading angle of the USV; $R_{\min}$ and $R_{\max}$ are the minimum and maximum turning radius, respectively, depending on the catamaran’s maneuvering performances [23,24].

#### 2.2.2. Controller Design

The concept of error dynamics is one of the most important skills for any controller design of motion control scenarios. First, we set the relation between the virtual catamaran and the own catamaran. For the path following problem, error variables are given as

(5a) $$x_{e}=x_{d}-x$$

(5b) $$y_{e}=y_{d}-y$$

(5c) $$\psi_{e}=\psi_{r}-\psi$$

(5d) $$z_{e}\;=\;\sqrt{x_{e}^{2}+}y_{e}^{2}$$

where $x_{d}$, $y_{d}$ denote the position of the virtual catamaran; $z_{e}$ is the position error; $\psi_{r}$ ($\psi_{r}\in \left(-\pi ,\;\pi \right]$) is the azimuth angle of the own catamaran relative to the virtual catamaran. It is necessary to distinguish the azimuth angle of the own catamaran ($\psi_{r}$) and the yaw angle of the virtual catamaran ($\psi_{d}$). In general, the azimuth angle of the USV is calculated as

(6) $$\;\psi_{r}\;=\;\{\begin{array}{cc} {}[1-0.5(1+\mathrm{sgn}(x_{e}))]\mathrm{sgn}(y_{e})\pi +\arctan\left(\frac{y_{e}}{x_{e}}\right), & z_{e}\neq 0\;\\ \psi_{d}\;, & z_{e}=0 \end{array}\;$$

where sgn ( ⋅ ) denotes a sign function with sgn (0)=1.

Moreover, the error variables can be rewritten as

(7) $$x_{e}=z_{e}\cos\;(\;\psi_{r}),\;y_{e}=z_{e}\cos\;(\;\psi_{r})$$

Based on Equations (1), (5), and (7), ${\dot{z}}_{e}$ and ${\dot{\psi }}_{e}$ are defined as

(8) $${\dot{z}}_{e}\;=\;{\dot{x}}_{d}\cos\;(\psi_{r})+{\dot{y}}_{d}\;\sin\;(\psi_{r})\;-u\;\cos\;\psi_{e}-v\;\sin\;\psi_{e}$$

(9) $${\dot{\psi }}_{e}=\;{\dot{\psi }}_{r}\;-\;r\;$$

(10) $${\dot{\psi }}_{r}\;=\;\frac{\left(\psi_{r}(t)\;-\;\psi_{r}(t-1)-360^{\circ }\;\right)}{\Delta t}$$

Based on the concept of error dynamics, a suitable control scheme should be implemented to achieve the goal of state convergence. The adaptive back-stepping method is applied to track the errors of surge, sway, and yaw motion. Therefore, the virtual control inputs for surge and yaw motion are defined as

(11) $$\alpha_{u}=\frac{k_{u1}(z_{e}-z_{m})+{\dot{x}}_{d}\cos\;\;\psi_{r}+{\dot{y}}_{d}\sin\;\psi_{r}-v\sin\psi_{e}}{\cos\;\psi_{e}}$$

(12) $$\alpha_{r}=k_{r1}\;\psi_{e}+\;{\dot{\psi }}_{r}$$

where $\alpha_{u}$ and $\alpha_{r}$ are the stabilizing functions of surge and yaw motion, respectively; $k_{u1}$, $k_{r1}\geq 0$ are the design parameters; $z_{m}\;=\exp\;(-0.054z_{e})$ is a small positive value. The control law ($\alpha_{u}$) will converge ($z_{e}-z_{m}$) to near zero. Own catamaran pursues the virtual catamaran with the help of ($z_{e}-z_{m}$) instead of $z_{e}$ [8,24].

Then, DSC skills [13] are employed to solve the problem of “explosion of complexity” at each backstepping step by introducing a new variable, $\beta_{i}$. Let $\alpha_{i}$ pass through a first-order filter $\beta_{i}$ with the time constant $\zeta_{i}$ to obtain $\beta_{i}$

(13) $$\zeta_{i}{\dot{\beta }}_{i}=-\beta_{i}+\alpha_{i},\;\beta_{i}\;(0)=\alpha_{i}\;(0)\;(i=\;u,\;r)$$

Define $y_{i}=\;\beta_{i}\;-\alpha_{i}$, then we have ${\dot{\beta }}_{i}\;=-\frac{y_{i}}{\zeta_{i}}$. With Equations (11)–(13), the time derivatives ${\dot{y}}_{u}$ and ${\dot{y}}_{r}$ are calculated as

(14a) $$\begin{array}{l} {\dot{y}}_{u}=-{\dot{\beta }}_{u}+{\dot{\alpha }}_{u}\\ \;=\;-\frac{y_{u}}{\zeta_{u}}+\;\frac{\partial \alpha_{u}}{\partial {\dot{x}}_{d}}{\ddot{x}}_{d}+\;\frac{\partial \alpha_{u}}{\partial x_{d}}{\dot{x}}_{d}+\;\frac{\partial \alpha_{u}}{\partial {\dot{y}}_{d}}{\ddot{y}}_{d}\;+\;\frac{\partial \alpha_{u}}{\partial y_{d}}{\dot{y}}_{d}\\ \;+\frac{\partial \alpha_{u}}{\partial x}\dot{x}+\;\frac{\partial \alpha_{u}}{\partial y}\dot{y}\;+\;\frac{\partial \alpha_{u}}{\partial v}\dot{v}\;+\frac{\partial \alpha_{u}}{\partial \psi }\dot{\psi }\;+\frac{\partial \alpha_{u}}{\partial \psi_{r}}{\dot{\psi }}_{r}\;\\ \;=\;-\frac{y_{u}}{\zeta_{u}}+Z_{u}(\cdot )\; \end{array}$$

(14b) $$\begin{array}{l} {\dot{y}}_{r}=\;-{\dot{\beta }}_{r}+{\dot{\alpha }}_{r}\\ \;=\;-\frac{y_{r}}{\zeta_{r}}+\;\frac{\partial \alpha_{r}}{\partial {\dot{\psi }}_{r}}{\ddot{\psi }}_{r}+\;\frac{\partial \alpha_{r}}{\partial \psi_{r}}{\dot{\psi }}_{r}\;+\frac{\partial \alpha_{r}}{\partial \psi }\dot{\psi }\;\\ \;=\;-\frac{y_{r}}{\zeta_{r}}+Z_{r}(\cdot )\; \end{array}$$

where $Z_{i}(\cdot )\;,\;i=u,\;r$ is the continuous function of the state variables [8].

By introducing the error variables of surge and yaw motion as $i_{e}=\;\beta_{i}\;-\;i$ instead of $i_{e}=\;\alpha_{i}\;-\;i$, i=u, r and the error dynamics ${\dot{u}}_{e}$ and ${\dot{r}}_{e}$ can be written as

(15a) $${\dot{u}}_{e}={\dot{\beta }}_{u}\;-\dot{u}={\dot{\beta }}_{u}\;-\;\left(W_{u}^{T}f_{u}+\frac{1}{m_{11}}\tau_{u}+\;\;\frac{1}{m_{11}}d_{w1}(t)\right)$$

(15b) $${\dot{r}}_{e}={\dot{\beta }}_{r}\;-\;\dot{r}={\dot{\beta }}_{r}\;-\;\left(W_{r}^{T}f_{r}+\frac{1}{m_{33}}\tau_{r}+\;\;\frac{1}{m_{33}}d_{w3}(t)\right)$$

(15c) $$W_{u}=[m_{22}/m_{11},\;d_{u1}/m_{11},\;d_{u2}/m_{11},\;d_{u3}/m_{11}]$$

(15d) $$W_{r}=[(m_{11}-m_{22})/m_{33},\;d_{r1}/m_{33},\;d_{r2}/m_{33},\;d_{r3}/m_{33}]$$

(15e) $$f_{u}={\left[\upsilon r,\;-u,\;-u\left|u\right|,\;-u^{3}\right]}^{T}$$

(15f) $$f_{r}={\left[u\upsilon ,\;-r,\;-r\left|r\right|,\;-r^{3}\right]}^{T}$$

where $m_{11}$, $m_{22}$, $m_{33}$, $d_{u2}$, $d_{u3}$, $d_{r1}$, $d_{r2}$, and $d_{r3}$ denote the unknown parameters of ship’s inertia and hydrodynamic damping coefficients. Then, the actual control inputs of own catamaran are correspondingly calculated as

(16a) $$\tau_{u}=m_{11}[k_{u2}u_{e}+{\dot{\beta }}_{u}+(z_{e}-z_{m})\cos\psi_{e}-{\hat{W}}_{u}^{T}f_{u}^{}+{\hat{d}}_{wu\;\;\max}\vartheta (\;u_{e})]$$

(16b) $$\tau_{r}=m_{33}[k_{r2}r_{e}+{\dot{\beta }}_{r}+\psi_{e}-{\hat{W}}_{r}^{T}f_{r}^{}+{\hat{d}}_{wr\;\;\max}\vartheta (r_{e})]$$

where $k_{u2}$, $k_{r2}\geq 0$ are the design parameters; ϑ( ⋅ ) is the smooth function satisfying Lemma 1 [11].

**Lemma** **1.** δ>0, *there exists a smooth function*ϑ( ⋅ ), *such that*ϑ( 0 ) = 0

(17) |ς|≤ςϑ(ς)+δ, ∀ς ∈ ℜ 

**Remark** **4.** *As in [11], the useful examples [8,22] satisfying Lemma 1 are as follows*

$$\vartheta (\varsigma )=\;\frac{1}{4\delta }\varsigma \;or\;\vartheta (\varsigma )=\tanh\;(\frac{\;\kappa \varsigma }{\delta })\;with\;\kappa =e^{-(\kappa +1)}\;$$

Then, along with Assumption 1, the adaptative law for parameters and estimated upper bound of disturbances are written as

(18a) $${\dot{\hat{W}}}_{u}=\gamma_{wu1}[-u_{e}f_{u}^{}-\gamma_{wu2}({\hat{W}}_{u}-{\hat{W}}_{u0})]\;$$

(18b) $${\dot{\hat{W}}}_{r}=\gamma_{wr1}[-r_{e}f_{r}^{}-\gamma_{wr2}({\hat{W}}_{r}-\;{\hat{W}}_{r0})]\;$$

(18c) $${\dot{\hat{d}}}_{wu\;\max}=\gamma_{dwu1}[u_{e}\vartheta (u_{e})-\gamma_{dwu2}({\hat{d}}_{wu\;\max}-{\hat{d}}_{wu\;\max0})]\;$$

(18d) $${\dot{\hat{d}}}_{wr\;\max}=\gamma_{dwr1}[r_{e}\vartheta (r_{e})-\gamma_{dwr2}({\hat{d}}_{wr\;\max}-{\hat{d}}_{wr\;\max0})]\;$$

*where* $\gamma_{wi1}$$\in {ℜ}^{n_{i}}$*,*i= u, r*denotes the positive definite matrix;*$\gamma_{wu1}$*,*$\gamma_{wu2}$*,*$\gamma_{dwu1}$*,*$\gamma_{dwu2}$*,*$\gamma_{dwr1}$*and*$\gamma_{dwr3}$*are the weighting factors;*$W_{u0}$*,*$W_{r0}$*,*$d_{wu\;\max\;0}$*,and*$d_{wr\;\max\;0}$*are the initial value of the design variables [8,11].*

## 3. Results (Stability Analysis)

### 3.1. Analysis Result

This section presents the stability analysis of closed-loop system using the Lyapunov theory. The main results are represented in the following Theorem 1.

**Theorem** **1.** *Consider an uncertain underactuated system (1) with Assumptions 1 to 3, the control law (16) and adaptive law (18); all the signals in the closed-loop system are satisfied as being semi-globally uniformly ultimately bounded (SGUUB) [8,23] if, for any Ω, a compact subset of $R^{n}$ and all $x(t_{0})=x_{0}\in \Omega$, there exists a μ>0 such that ‖x(t)‖<μ [38]*.

**Proof.**  Introducing the Lyapunov function candidate as

(19) $$\begin{array}{l} V=\;\frac{1}{2}({\left(z_{e}-z_{m}\right)}^{2}+\psi_{e}^{2}+y_{u}^{2}+y_{r}^{2}+u_{e}^{2}\;+r_{e}^{2}\\ +{\tilde{W}}_{u}^{T}\gamma_{wu1}^{-1}\;{\tilde{W}}_{u}^{}\;+\;{\tilde{W}}_{r}^{T}\gamma_{wr1}^{-1}\;{\tilde{W}}_{r}^{}+\gamma_{dwu1}^{-1}\;{\tilde{d}}_{wu\;\max}^{2}\;+\;\gamma_{dwr}^{-1}\;{\tilde{d}}_{wr\;\max}^{2}) \end{array}$$

Its time derivatives can be written as

(20) $$\begin{array}{ll} \dot{V}=\; & (z_{e}-z_{m}){\dot{z}}_{e}+\psi_{e}^{}{\dot{\psi }}_{e}^{}+y_{u}^{}{\dot{y}}_{u}^{}+y_{r}^{}{\dot{y}}_{r}^{}+u_{e}^{}{\dot{u}}_{e}^{}+r_{e}^{}{\dot{r}}_{e}^{}\\ & +{\tilde{W}}_{u}^{T}\gamma_{wu1}^{-1}\;{\dot{\hat{W}}}_{u}^{}\;+\;{\tilde{W}}_{r}^{T}\gamma_{wr1}^{-1}\;{\dot{\hat{W}}}_{r}^{}+\gamma_{dwu}^{-1}\;{\tilde{d}}_{wu\;\max}^{}{\dot{\hat{d}}}_{wu\;\max}^{}+\gamma_{dwr}^{-1}\;{\tilde{d}}_{wr\;\max}^{}{\dot{\hat{d}}}_{wr\;\max}^{}\\ & \leq \;-\;(k_{u1}-2\;){\left(z_{e}-z_{m}\right)}^{2}-(k_{r1}-2\;)\psi_{e}^{2}\\ & -\sum_{i=u,\;r}(\frac{y_{i}^{2}}{\zeta_{i}}-\frac{y_{i}^{2}}{4}-\frac{G_{i}^{2}y_{i}^{2}}{2b_{1}}+\frac{i_{e}^{2}}{2}+\;\;i_{e}(W_{i}^{T}f_{i}-d_{wi}-\tau_{i})+\\ & {\tilde{W}}_{i}^{T}\gamma_{wi1}^{-1}\dot{\hat{W}}+\;\gamma_{dwi1}^{-1}{\tilde{d}}_{wi\max}{\dot{\hat{d}}}_{wi\max})+b_{1} \end{array}$$

By substituting (16a,b), (18a–d) into (20), we obtain

(21) $$\begin{array}{ll} \dot{V}\leq & \;-\;(k_{u1}-2\;){\left(z_{e}-z_{m}\right)}^{2}-(k_{r1}-2\;)\psi_{e}^{2}\\ & -\;\sum_{i=u,\;r}(\frac{y_{i}^{2}}{\zeta_{i}}-\frac{y_{i}^{2}}{4}-\frac{G_{i}^{2}y_{i}^{2}}{2b_{1}}+\left(k_{i2}-\frac{1}{2}\right)i_{e}^{2}-\;i_{e}{\tilde{W}}_{i}^{T}f_{i}\\ & +d_{wi\;\max}\left|i_{e}\right|-\;d_{wi\;\max}i_{e}\vartheta (i_{e})-\;{\tilde{d}}_{wi\;\max}i_{e}\vartheta (i_{e})\\ & +{\tilde{W}}_{i}^{T}\gamma_{wi1}^{-1}\dot{\hat{W}}+\;\gamma_{dwi1}^{-1}{\tilde{d}}_{wi\max}{\dot{\hat{d}}}_{wi\max})+b_{1}\\ \leq & -\;(k_{u1}-2\;){\left(z_{e}-z_{m}\right)}^{2}-(k_{r1}-2\;)\psi_{e}^{2}\\ & -\;\sum_{i=u,\;r}(\frac{y_{i}^{2}}{\zeta_{i}}-\frac{y_{i}^{2}}{4}-\frac{G_{i}^{2}y_{i}^{2}}{2b_{1}}+\left(k_{i2}-\frac{1}{2}\right)i_{e}^{2}\\ & +\frac{\gamma_{wi2}}{2\gamma_{wi1}}{\tilde{W}}_{i}^{T}\gamma_{wi1}^{-1}{\tilde{W}}_{i}^{}+\;\frac{\gamma_{dwi1}\gamma_{dwi2}}{2}\gamma_{dwi1}^{-1}{\tilde{d}}_{wi\max}^{2})+b_{2}\\ \leq & -a_{1}V+b_{2} \end{array}$$

where $b_{1}$, $G_{i}$, $a_{1}$, and $b_{2}$ are the positive constants satisfying

$$\left|Z_{i}(\;\cdot \;)\right|\leq G_{i},\;i=\;u,\;r$$

$$a_{1}=\min\{\frac{\gamma_{wu2}}{2\kappa_{\max}(\gamma_{wu1})},\;\frac{\gamma_{wr2}}{2\kappa_{\max}(\gamma_{wr1})},\;\frac{\gamma_{dwu1}\gamma_{dwu2}}{2},\;\frac{\gamma_{dwr1}\gamma_{dwr2}}{2}\}$$

$$b_{2}=\sum_{i=u,\;r}\left(\delta_{i}d_{wi\;\max}+0.5\left(\gamma_{wi2}{\left\|W_{i}-{\hat{W}}_{i0}\right\|}^{2}+\gamma_{dwi2}{\left(d_{wi\;\max}-{\hat{d}}_{wi\;\max0}\right)}^{2}\right)\right)+b_{1}$$

Then, we get $V(t)\leq \;b_{2}/2a_{1}+\left(V(0)-b_{2}/2a_{1}\right)\exp(-2a_{1}t)$ by integrating Equation (21). V(t) is bounded satisfying $\lim_{t\to \infty }V(t)\leq \;b_{2}/2a_{1}$. All the signals in the closed-loop system can be guaranteed to be SGUUB [8,23,38]. □

### 3.2. On the Passive-Boundedness of Sway Motion

This section discusses the passive-boundedness of sway velocities in Assumption 3. Passive-boundedness means that the sway speed is bounded in cases where all other variables are bounded [11]. This is related to hydrodynamic damping, which interferes with the motion of marine catamarans. Since underactuated marine catamarans have no control input with respect to sway motion, the passive-boundedness should be defined with the inclusion of the sway disturbance. The sway dynamics in Equation (1b) is considered to be

(22) $$\dot{v}=W_{v}^{T}f_{v}(\dot{\eta },\;\eta )+\frac{1}{m_{22}}d_{wv}(t)$$

with

$$W_{v}=[m_{11}/m_{22},\;d_{v1}/m_{22},\;d_{v2}/m_{22},\;d_{v3}/m_{33}]$$

$$f_{v}={\left[ur,\;-v,\;-v\left|v\right|,\;-v^{3}\right]}^{T}$$

where $d_{v1}$, $d_{v2}$, and $d_{v3}$ refer to the unknown parameters of hydrodynamic damping and nonlinear damping terms. Considering the Lyapunov candidate as $V_{v}=0.5v^{2}$, then its time derivatives can be written as

(23) V˙v=1m22(dv1+dv2|v|+dv3)v2+vm22(dwv−m11ur)≤1m22(dv1+dv2|v|+dv3)v2+ξ2m22= −2(dv1+dv2|v|+dv3−0.25)Vv+ξ2m22

where ξ is a positive constant for the upper bound satisfying $\xi \geq \;\left|d_{wv}-m_{11}ur\right|$. If $\left|v\right|\geq \left|d_{wv}-m_{11}ur\right|/\;{\left(d_{v1}-0.25\right)}^{0.5}$, then ${\dot{V}}_{v}\;\leq \;0$. Thus, v satisfies being passive-bounded and, further, being uniformly ultimate bounded [8,11,22].

## 4. Discussion

### 4.1. Williamson Turning Reports of the Existing Marine Vessels

**Figure 3:**
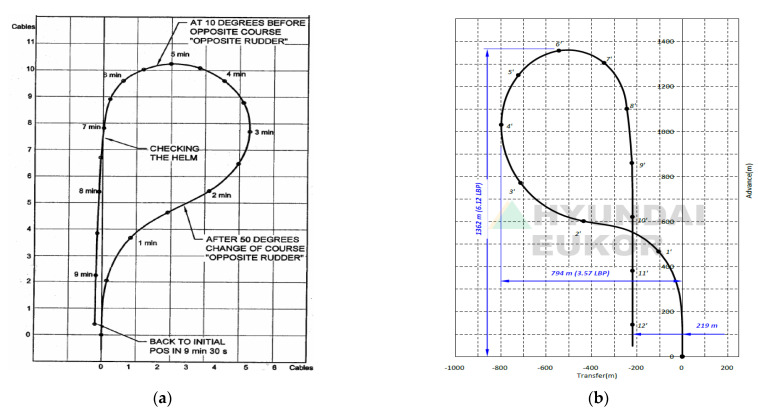
Williamson turning reports of merchant ships: (**a**) VLCC ($L_{pp}$= 349.8 m,∇ = 355,600 m^3^) under loading conditions; (**b**) 8100 unit roll_on roll_off car carrier ($L_{pp}$ = 222.4.m,∇ = 29,917 m^3^) under ballast conditions.

**Figure 4:**
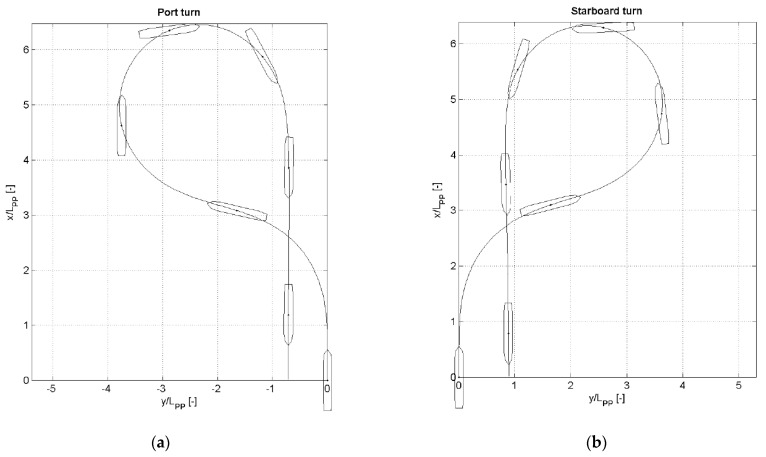
Williamson turning reports of a training ship (T/S) Segero ($L_{pp}$= 120 m,∇ = 9122.2 m^3^) governed by Mokpo National Maritime University: (**a**) port turn; (**b**) starboard turn.

First of all, this section addresses the fact that it is difficult for existing marine vessels (VLCCs, car carriers, and training ships) to return to their original course via the Williamson turning method. Since it is based on a destroyer, which has two controllable pitch propellers and two main engines, it is necessary to modify the traditional Williamson method. For example, the Williamson turning reports for a VLCC ($L_{pp}$ = 349.8 m, ∇ = 355,600 m^3^) with under loaded conditions [39], an 8100 unit roll_on roll_off car carrier ($L_{pp}$ = 222.4 m, ∇ = 29,917 m^3^) under ballast conditions [20], and a training ship (T/S) Segero ($L_{pp}$ = 120 m, ∇ = 9122.2 m^3^) governed by Mokpo National Maritime University in Figure 3a,b, and Figure 4, respectively, where $L_{pp}$ and ∇ refer to the length between perpendiculars and displacement, respectively. The degree of deviation from the original course can be seen in Figure 3 and Figure 4. Clearly, it can be observed that it is difficult for conventional marine vessels to return to their initial point. Thus, this paper focuses on returning an underactuated catamaran to the exact site of casualties by adjusting the rate of turn rather than the rudder orders.

### 4.2. Experimental Turning Test by Remote Control

The maneuvering characteristics represent the steady turning radius, advances, transfer, tactical diameter quantitatively. However, many reports of path following control overlook the maneuvering characteristics and it tends to select the existing model in the previous publications [2,3,5,8,9,11,22,23,24]. This paper considers a practical catamaran having two electric propellers for recovery maneuvers. To confirm the maneuvering conditions for the model ship, the sea experiment has done based on the remote control. Actually, it is difficult to predict the maneuvering characteristics of the catamaran by the model test due to the lack of specific steering information and roll interaction [40]. However, the main purpose of the experiment is to advance the remote DC propulsion system, communication system, and turning abilities. Additionally, a concrete tracking algorithm was not adopted in this step.

**Figure 5:**
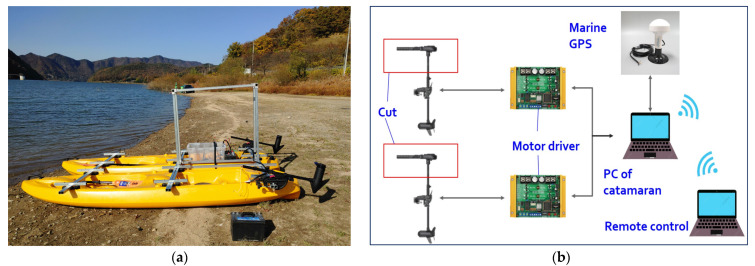
System structure of unknown underactuated catamaran: (**a**) side view of two pontoons and electrical propellers; (**b**) control structure for remote control inshore.

**Figure 6:**
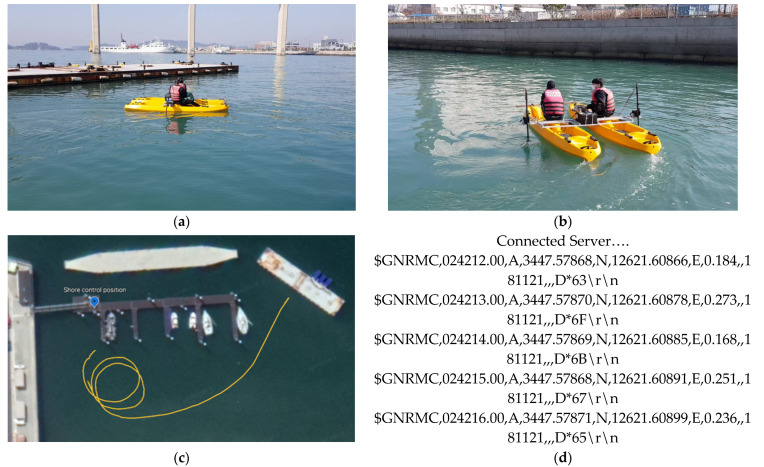
Remote control test of maneuvering conditions with two men on board: (**a**) the boat moves to the test area; (**b**) the USV exhibits bounded sway motion during port turning by means of yaw control; (**c**) sea trajectory consisting of two circles with 17 m diameters and a straight line; (**d**) data information (position, SOG, heading in order, etc.) obtained from marine GPS sensors.

Figure 5 depicts the hull of two pontoons with two propellers and the construction of the control system. A marine GPS sensor constantly records the information of position, direction, over ground speeds, etc. Figure 6a–d show the test area (calm sea), port turning situation, trajectory, and data information, respectively. The tactical diameter and turning radius ($R_{arc}$) are approximately marked as 21 m and 10 m, respectively. The turning radius of steady states is proportional to the ship’s length and inversely proportional to the angle of the rudder actuator [41]. The experimental results can be compared to the maneuvering characteristics of Marine-class vessels ($L_{OA}$= 171.8 m,∇=18,541 m^3^) [6], where $L_{OA}$ refers to the overall length. It can be observed that the catamaran has sufficient displacement, high propulsion, and maneuvering conditions with two men on board and two heavy batteries, etc. This weight will be calculated as the additional payloads in Section 4.3.

### 4.3. Main Parameters for Simulation

This section addresses the main parameters of the catamaran shown in Figure 5a. The test model consists of two pontoons and other equipment on the superstructure. Table 1 presents the values of a single pontoon and the full catamaran. We can assume that there is some kind of cargo or luggage on deck and in the hold spaces. The additional payloads resulted in the movements of the CG. The inertia matrix can be calculated as $I_{z}=I_{z}^{CG}+m(x_{g}^{2}+y_{g}^{2})$, where $x_{g}$, $y_{g}$ denote the corrected location in CG due to the payloads; $I_{z}^{CG}=mR_{66}$ is the moment of inertia about CG where $R_{66}=0.25L$ is the radius of gyration with respect to the CG [6]. Moreover, the added mass coefficients represent the amount of fluid accelerated with the catamaran. The particles of the fluid accelerate to some extent as the catamaran moves [42]. The hydrodynamic added mass in the surge ($X_{\dot{u}}$) can be given as $-2.7\rho \;\nabla^{5/3}/L^{2}$ [43,44], where ρ=1025 (kg/m^3^) is the density of the sea water. The added mass coefficients in the sway ($Y_{\dot{v}}$) and yaw ($N_{\dot{r}}$) can be approximately calculated as −1.5*m* and $-1.7I_{z}$, respectively [41,44]. The other linear damping terms can be seen in Table 1 and Refs. [6,44]. The hydrodynamic forces and moments for the Munk moment and resistances are modeled as a nonlinear function of velocity and acceleration ($\upsilon ,\;\dot{\upsilon }\;$) and the Euler angles of η [40]. These functions are defined in Equations (15e) and (15f). It is not necessary to express all of the hydrodynamic derivatives for the practical catamaran; it is acceptable to implement a nonlinear control scheme, as reported in Refs. [8,9,11,22]. Thus, the unknown parameters in the nonlinear parts are set approximately to be $d_{i2}=0.2d_{i1}$, $d_{i3}=0.1d_{i1}$(i=u, r), with ± 15% of the value of the linear damping [9] according to the adaptation laws (18a, b), and $d_{v2},\;d_{v3}=0.2d_{u1}$ for more dynamical responses in sway motion.

### 4.4. Predefined Recovery Paths

In the path following problem, the virtual catamaran generates predefined recovery routes on the basis of the guiding principle. Originally, a ship’s sailing route consists of many way-points (latitude and longitude information). The proposed guiding algorithm only uses the turning rates of the virtual catamaran, which helps to reduce the effort of determining recovery routes. Several publications have been focused on similar routes, restricted only to port turning [8,9,11,22]. Therefore, we designed unusual recovery routes that include continuative turning to the port and starboard side, as well as straight courses to return to the original departure point. At first, the Williamson route was modified to return the initial point using Equation (24). Additionally, one of the complex recovery routes, including double circles and an S-shaped trajectory (consecutive turning in the inbound and outbound directions), is demonstrated by Equation (25). Then, the catamaran will arrive near the initial point in order to rescue the MOB.

(24) $$r_{d}=\left\{ \begin{array}{lll} -\exp(0.005t/200), & 0\leq t<20; & \\ -0.05, & 2\;0\leq t<50; & starboard\;turn\\ 0.05, & 50\leq \;t<144.5\;; & port\;turn\\ 0, & 144.5\leq \;t<231; & \end{array}\right.$$

(25) $$r_{d}=\left\{ \begin{array}{lll} \exp(0.01t/200), & 0\leq t<10; & \\ 0\;, & 10\leq t<30; & 1st\;circle\\ 0.05, & 30\leq \;t<150\;; & \\ \exp(0.005t/300), & 150\leq \;t<210; & 2nd\;circle\\ 0.05, & 210\leq \;\;t<350; & \\ -0.05, & 350\leq \;t<437; & 3rd\;circle\\ 0, & 436\leq \;t<574; & \end{array}\right.$$

The initial conditions corresponding to the MOB are set to [x(0), y(0), ψ(0), u(0), v(0), r(0)] = [−120 m, 15 m, −0.15 rad, 0 m/s, 0 m/s, 0 rad/s]. The values of the control parameters are listed in Table 2. Above all, if readers wish to change the model ship, the parameter $\gamma_{wr1}$, which relates to the inertia matrix, should be adjusted.

Moreover, environmental disturbances interfere with the computational calculation of derivatives in the backstepping methods. Even though the model ship is a small-sized craft, the surge speed of the virtual catamaran is set to $u_{d}=6$ m/s for the consideration of sufficient forward speeds. In the case of decreasing the parameter $u_{d}$, the turning radius ($R_{arc}$) decreases. In addition, a time history of the intentional disturbance is generated in order to simulate the control scheme in a simplified manner. As reported in previous works [33,34,35,36], despite the sinusoidal disturbances, the complex responses of marine catamarans due to their nonlinear dynamics characteristics are shown. Thus, time-varying disturbances acting on the own catamaran are considered as follows:

(26) $$d_{w}=\{\begin{array}{c} d_{w1}=0.5\sin(0.2t)+0.15\cos(0.5t)+0.5\\ d_{w2}=0.3\cos(0.4t)+0.3\cos(0.1t)+0.4\\ d_{w3}=0.8\sin(0.3t)+0.4\cos(0.5t)+1.5 \end{array}$$

The above disturbances are not realistic in practice; however, they are effective for showing the distances of deviations in the surge, sway, and yaw directions during path following. The sway disturbance $d_{w2}$ has a strong effect on the computational success and course-keeping ability. Moreover, considerable degrees of yaw rate can be expected, owing to the magnitude of yaw disturbance.

### 4.5. Simulation Results

**Figure 7:**
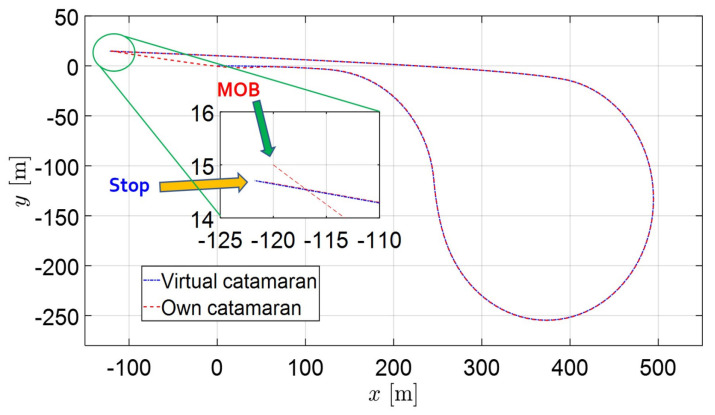
Path following control results of recovery maneuvers of the improved Williamson method by means of turning rate and its magnified zoom of a part of the trajectory in calm sea (without disturbances).

**Figure 8:**
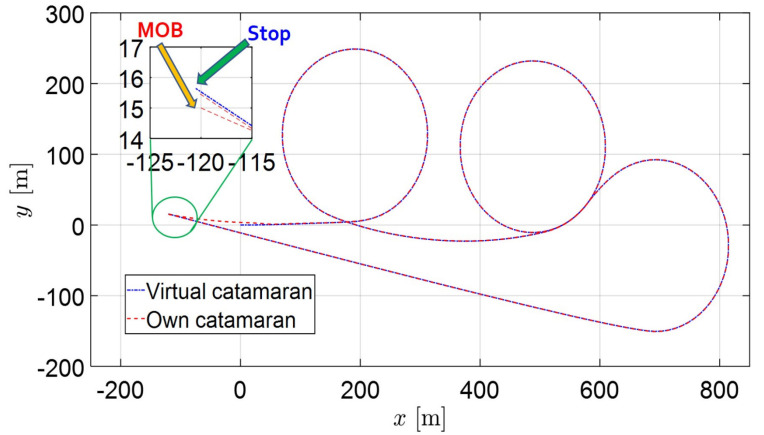
Path following control results of recovery maneuvers of complex routes including three circles, a straight line, and successive opposite turning in severe circumstances (with disturbances).

**Figure 9:**
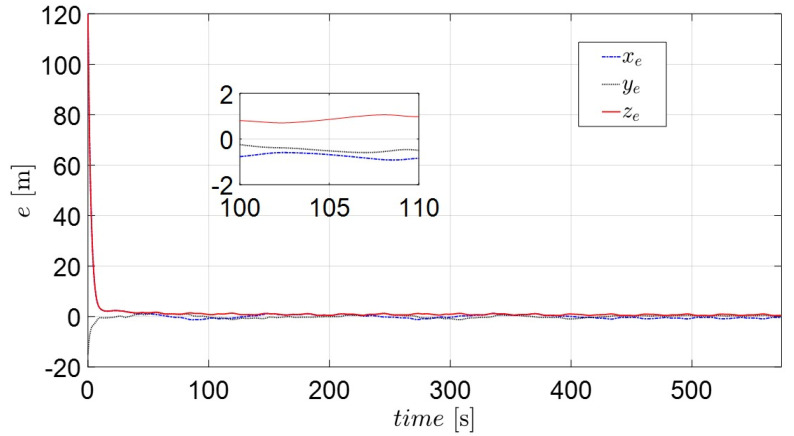
The position and orientation error variables ($x_{e},\;y_{e},\;z_{e}$) of the actual catamaran.

**Figure 10:**
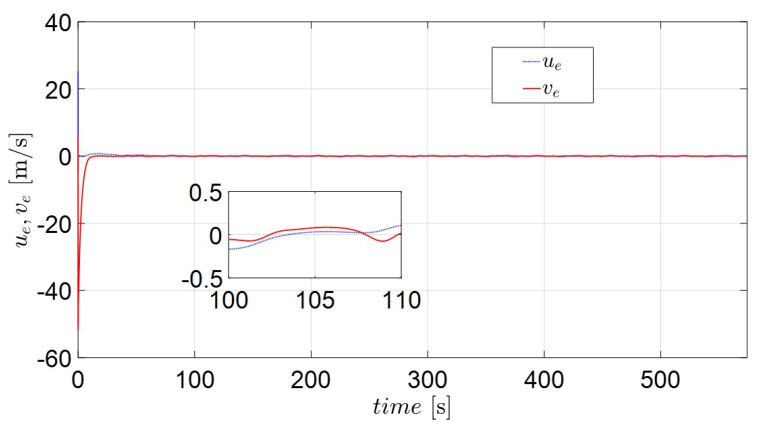
The position and orientation error variables ($u_{e},\;v_{e}$) of the actual catamaran.

**Figure 11:**
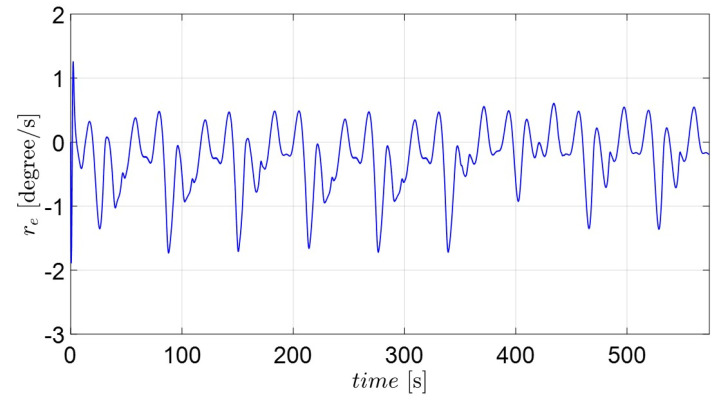
The position and orientation error variables ($\;r_{e}$) of the actual catamaran.

**Figure 12:**
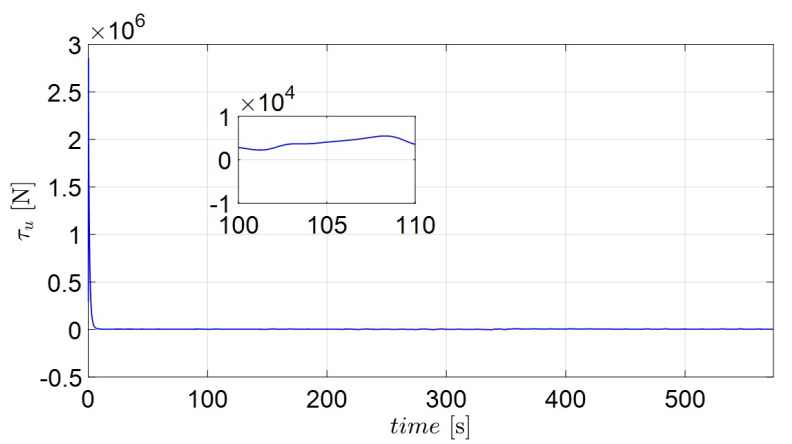
Corresponding control burden of surge force ($\tau_{u}$).

**Figure 13:**
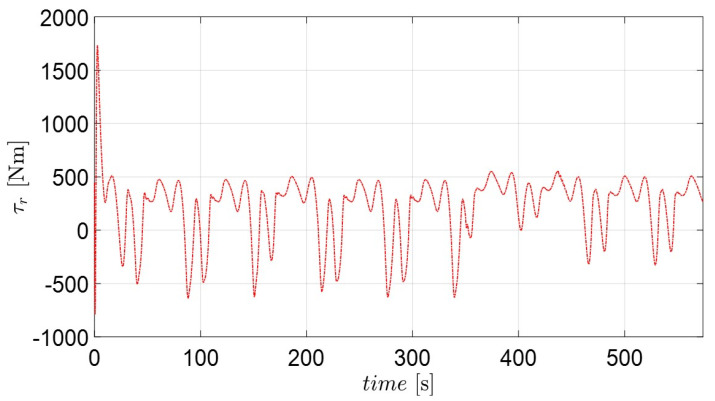
Corresponding control burden of yaw moment ($\tau_{r}$).

In this section, two recovery routes are considered for the control simulation. The first is Williamson turning in calm sea (without disturbances), the other is a more complex routes under disturbances. Several pictures are shown to demonstrate the performance of path following control from Figure 7, Figure 8, Figure 9, Figure 10, Figure 11, Figure 12 and Figure 13. The trajectories of the virtual catamaran (blue dashed-dotted line) followed by the actual own catamaran (red dashed line) are depicted in both Figure 7 and Figure 8, which illustrate the autonomous recovery maneuvers.

Figure 7 depicts the completion of the Williamson turning to within 1 m, as seen in the magnified zoom of a part of the trajectory. Figure 8 presents the recovery trajectories for complex routes, including three circles, a straight line, and successive opposite turning under external disturbances. Even though the routes and environmental situation become more severe, the catamaran arrives within 1 m, as seen in the magnified figure. The own catamaran successfully returns to the departure way-point. In this path, the successive turning may cause a calculation problem in differentiating the error variables. Under three directional disturbances and forward speed, the combined port and starboard maneuvering of the own catamaran easily causes a break of simulation, because this guiding algorithm is only based on the turning rate of the virtual catamaran to determine the routes. This means that the own marine catamaran maintains her position (way-points) on the sea by means of turning rate only. This looks like a difficult task for the helmsman and marine officers in traditional marine vessels, but unmanned catamarans may overcome the same situation in the near future.

Figure 9 and Figure 11 depict the position and orientation errors of $x_{e}$, $y_{e}$, $z_{e}$, $u_{e}$, $v_{e}$ and $r_{e}$ for the complex routes. Most error variables converge to near zero, with the exception of the yaw variables ($r_{e}$). The error of the diagonal line ($z_{e}$) in Figure 9 shows that the own catamaran closely follows the virtual catamaran at intervals of 1 to 2 m. This means that the adaptive backstepping control methods compensate for the effect of environmental disturbances during the entire sailing time. The errors of surge and sway velocity ($u_{e}$, $v_{e}$) quickly decrease to near zero under any initial condition in Figure 10, which demonstrates what was stated in the discussion of the passive-boundedness of sway motion, even though sway disturbance continuously excites the own catamaran. Unfortunately, owing to the yaw disturbance as well as the consecutive turning process, it is difficult for the error of yaw velocity ($r_{e}$) to converge to zero, as seen in Figure 11. This means that the own catamaran has good performance in path tracking; however, it shows the difficulty of maintaining a steady course under environmental disturbances. Since the error of yaw rate still has an oscillating features from 436 s (straight course) on the basis of Equation (25), this is regarded as the threshold of control scheme. It is for this reason that the trajectories converge to an invariant set rather than the equilibrium [22]. However, the maneuverability and stability of the underactuated catamaran can be regarded as acceptable considering the severe conditions.

Surge and yaw control loads are plotted in Figure 12 and Figure 13. The yaw moment ($\tau_{r}$) fluctuates more than the surge force ($\tau_{u}$). The result of $\tau_{r}$ can be improved by means of the parameter $k_{r1}$. However, an actuator may be damaged due to frequent control actions. Therefore, a robust control method such as adaptive super-twisting sliding mode control [35,45] can be substituted to suppress the control activities. Anyway, a trade-off should be considered when choosing the appropriate values of control parameters. Finally, it can be observed that the own catamaran under three directional disturbances successfully follows the predefined recovery routes and returns to the position of the casualties (MOB) using the proposed adaptive backstepping methods.

## 5. Conclusions

This paper investigated a model-based approach to path following control for recovery maneuvers with unknown dynamics of an underactuated practical catamaran. The goal of performing recovery maneuvers to within 1 m of the MOB was accomplished using an adaptive backstepping controller including DSC techniques. This study extends the application of control developments presented in many similar publications using existing known models to an unknown real model in practice. On the basis of this investigation, the following conclusions can be drawn.

(1) The main difficulty of model-based path following control for predicting the hydrodynamic derivatives of a practical catamaran was solved by the approximate calculation of mass, added mass, and linear damping in a diagonal matrix of a 3DOF system. Thus, readers may apply to their own model instead of models presented in previous publications. However, this paper did not consider any artificial intelligence methods, such as neural network or reservoir computing, for predicting the unknown restoring term.
(2) The second key problem of differential calculation for an underactuated model in the case of complex reference trajectories, including three circles, a straight line, and successive opposite turning under severe disturbances, was solved, achieving fast convergence and robust stability. This result may apply to the consecutive surveillance of USVs in the coastal area with complex routes.
(3) As an experimental test performed by remote control, the small craft with payloads at high speeds showed the existence of $m_{23}$ in practice. Even though this paper employs a diagonal matrix with unknown nonlinear terms, the problem of sway force per yaw moment (equals to sway/yaw added mass) needs to be tackled in turning cases with high speeds in both experimental testing and theory, without hydrostatic or restoring force terms. However, the unknown nonlinear parts lead to nonlinear dynamic phenomena that are highly essential in marine vessels under severe circumstances.

Finally, it can be concluded that the underactuated practical catamaran successfully tracked the recovery routes under environmental disturbances.

## Figures and Tables

**Table 1:** Main parameters of catamaran.

	Parameters	Values
One pontoon	$L_{OA}$	3.6 m
	Beam	0.77 m
	Depth	0.265 m
Catamaran	$I_{z}$	45.65 kg m^2^
	$X_{\dot{u}}$	−14.61 kg
	$Y_{\dot{v}}$	−82.5 kg
	$N_{\dot{r}}$	−77.61 kg m^2^ s^−1^
	$X_{u}$	77.55 kg s^−1^
	$Y_{v}$	0 kg s^−1^
	$N_{r}$	246.51 kg m^2^s^−1^
	$m_{11}$	69.6 kg
	$m_{22}$	137.5 kg
	$m_{33}$	123.25 kg

**Table 2:** Control parameters.

Notation	Values	Notation	Values	Notation	Values
$k_{u1}$	0.2	$\gamma_{wu1}$	0.05	$\gamma_{dwu1}$	1
$k_{u2}$	20	$\gamma_{wu2}$	0.2	$\gamma_{dwu2}$	2
$k_{r1}$	1.8	$\gamma_{wr1}$	0.3	$\gamma_{dwr1}$	1
$k_{r2}$	120	$\gamma_{wr2}$	0.2	$\gamma_{dwr2}$	0.2

## Data Availability

Not applicable.
